# Investigating the efficacy of non-invasive vagus nerve stimulation in treating drug-resistant epilepsy

**DOI:** 10.6026/973206300211551

**Published:** 2025-06-30

**Authors:** Daleena Prathiba Madda, Sruthi Vempatapu, Sasikanth Kanaganuru

**Affiliations:** 1 Department of General Medicine, New Vara Hospital, Hyderabad, Telangana, India; 2 Department of Emergency Medicine, Yashoda hospitals, Hyderabad, Telangana, India; 3 Department of General Medicine, Apollo Hospitals, Hyderabad, Telangana, India

**Keywords:** Non-invasive vagus nerve stimulation, drug-resistant epilepsy, seizure frequency, quality of life, neuromodulator, alternative therapy

## Abstract

Drug-resistant epilepsy (DRE) affects approximately 30% of individuals with epilepsy who do not respond to antiepileptic drugs. This randomized controlled trial evaluated the efficacy of non-invasive vagus nerve stimulation (nVNS) in 120 DRE patients. Over 12 weeks, the intervention group showed a 45% reduction in seizures and a 30% improvement in quality of life, both statistically significant. Minor side effects included headaches and neck discomfort. nVNS appears to be a safe and effective adjunctive therapy for managing DRE.

## Background:

Chronic neurological condition Epilepsy results in unprovoked recurrent seizures which negatively affect the lifestyle quality of those affected individuals. Although antiepileptic drugs (AEDs) have advanced there remains a population of 30 percent of epilepsy patients experiencing seizures which qualifies their condition as drug-resistant epilepsy (DRE) according to studies [1]. The on-going presence of seizures after achieving maximum drug control requires new therapeutic methods because current treatments are insufficient [2]. Researchers have identified non-invasive vagus nerve stimulation (nVNS) as an emerging and promising therapy because it successfully decreases seizures yet requires no surgery [3]. The original Vagus nerve stimulation (VNS) devices operated through implantable device therapy before leading to surgical insertion of a pulse generator attached to the vagus nerve [4]. The use of handheld stimulators to deliver electrical impulses to cervical vagus nerve represents a more accessible and lower-risk alternative approach [5]. Neuronal excitability changes occur when afferent nerve signals reach the brainstem to operate on subcortical and cortical networks that produce seizures [6]. Scientific research shows that nVNS reduces abnormal brain activity and improves brain inhibition to decrease seizures [7, 8]. Multiple clinical trials together with observational studies established nVNS produces beneficial results through its ability to decrease seizure occurrences coupled with better quality of life markers [9, 10]. The inconsistent study findings stem from various study factors such as designs and patient demographics along with stimulation protocols thus scientists need to develop standardized nVNS application guidelines [11]. Therefore, it is of interest to investigate the clinical performance of non-invasive vagus nerve stimulation as it relates to seizure control and patient lifestyle improvements in cases of drug-resistant epilepsy.

## Materials and Methods:

## Study design and participants:

One hundred twenty patients who met the International League against Epilepsy (ILAE) DRE diagnosis criteria received recruitment at various neurology clinics throughout multiple Centers in Vijayawada, Andhra Pradesh. The study included patients who could not reach seizure control goals after a twelve-month trial period of using at least two correctly selected medications at optimal doses which were tolerated. The study exclusion process eliminated participants who underwent vagus nerve surgical procedures and presented psychiatric disorders requiring hospital admission and patients with major heart diseases and pregnant or breastfeeding women.

## Randomization and blinding:

No randomization process occurred since participants divided in half through a 1:1 distribution with sixty participant's active nVNS users and sixty participants in the sham stimulation group. A computer-generated randomization list generated the allocation concealment by deploying randomization procedures. All participants and investigators maintained their lack of knowledge about the assigned treatments during the entire duration of the research period.

## Intervention:

The active nVNS portable device for cervical vagus nerve stimulation served as the intervention for the study participants. The device required bilaterial application on the neck area for 2-minute sessions performed three times each day for 12 weeks. A built-in program within the device delivered electrical pulses with pre-programmed parameters that matched the therapeutic requirements of nVNS therapy. Participants in the control group used a matching device that released no electrical stimulation but imitated both visual aspects and operational protocols of the functional device. This ensured effective blinding of participants to their group allocation.

## Outcome measures:

The main effect assessed seizure frequency change throughout the twelve-week study period from the baseline assessment. The study evaluated QoL by using the Quality of Life in Epilepsy Inventory (QOLIE-31) assessment as one of the secondary outcome measures in addition to recording any adverse reactions stemming from the therapy. The research team conducted assessments at start, week 6 and final endpoint at week 12.

## Data collection and analysis:

During the follow-up appointments patients presented their daily seizure diaries that recorded seizure occurrence. The Quality of Life in Epilepsy Inventory (QOLIE-31) questionnaires served to gather quality of life scores from patients completing them. Study investigators collected adverse effect information from patients alongside clinical healthcare observations. The statistical evaluation occurred through SPSS software version 26. The research design included paired t-tests to measure changes inside each group across the specified measurement points and repeated measures ANOVA to assess differences in seizure count and QoL scores between groups during baseline, week 6 and week 12 evaluation periods.

## Results:

The research enrolled 120 patients into the study while separating them into two groups of 60 for each independent group. The research included 120 participants who maintained their partipation until the 12-week checkup without any participant leaving the study.

## Seizure frequency reduction:

During the 12-week trial the patients in the experimental group exhibited stronger outcomes featuring seizure reduction compared to participants in the control group. Research findings showed a 45% decrease (p < 0.01) with the intervention group averaging 8.7 ± 3.9 seizures per month at 12 weeks although the baseline measurement had been 15.8 ± 4.3 seizures per month. The control group participants recorded minimal seizure reduction from 16.1 ± 4.5 seizures per month at baseline to 14.8 ± 4.1 seizures per month whereas the researchers found this result statistically insignificant (p = 0.12) (Table 1 - see PDF).

## Quality of life improvement:

Quality of life (QoL) scores measured by the QOLIE-31 inventory showed significant improvement in the intervention group compared to the control group. The intervention group experienced an increase in QoL scores from 55.4 ± 8.2 at baseline to 72.0 ± 7.5 at 12 weeks, reflecting a 30% improvement (p < 0.05). The control group showed a negligible improvement from 54.9 ± 8.0 to 57.6 ± 7.8 (5% improvement, p = 0.28) (Table 2 - see PDF).

## Adverse effects:

Mild adverse effects were reported by 10% of participants in the intervention group, primarily consisting of headache and transient neck discomfort. No significant adverse effects were reported in the control group. The findings of this study suggest that non-invasive vagus nerve stimulation significantly reduces seizure frequency and improves the quality of life in patients with drug-resistant epilepsy compared to sham stimulation.

## Discussion:

A research study utilizing randomization revealed that non-invasive vagus nerve stimulation (nVNS) creates both seizure reduction and quality of life improvement in drug-resistant epilepsy patients (DRE). Patients in the intervention group achieved a 45% reduction in seizure frequency which the control group did not match despite their 8% decrease. Research by previous studies has shown positive effects of vagus nerve stimulation for epilepsy patients refractory to other treatments [1, 2]. Vagus nerve stimulation (VNS) has shown sustained efficacy and safety over the past 30 years as an adjunctive treatment for drug-resistant epilepsy, offering seizure reduction and improvement in comorbid conditions such as depression and anxiety [3]. The activation of vagus nerve leads to improved inhibitory neurotransmitter which in turn reduces excessive cortical activity thus decreasing the number of seizures [4]. The neuroimaging evidence indicates that after nVNS therapy occur decreases in cortical hyper-excitability [5]. Non-invasive approaches for vagus nerve stimulation have gained popularity because their safety characteristics along with user-friendly design outperform implanted medical devices [6]. The nVNS devices which transmit electrical impulses through the skin bypass surgical implantation thus create minimal risk factors for surgical complications as well as postoperative morbidity [7]. The advantage of nVNS treatment makes it suitable for patients who do not want to undergo conventional invasive medical procedures [8]. Various former studies presented conflicting findings about nVNS effectiveness in DRE because of distinction in stimulation protocols and patient demographics along with research methodology [9, 10]. The findings from Meta-analysis conducted by Redgrave *et al.* (2018) demonstrated that non-invasive stimulation led to a 30% drop in seizure frequencies yet the wide range of study consistency persisted at 11%. The recorded 45% reduction matches the higher metrics released in past research because of refined stimulation settings together with diligent participant compliance. Our study demonstrates that patients in the intervention group experienced better quality of life as a major research finding. The treatment of chronic epilepsy causes substantial damage to physical as well as emotional and social functioning of patients even when seizure frequency decreases [12]. The substantial QoL improvement in our study proves that nVNS delivers additional advantages over seizure control alone since it provides patients with reduced anxiety and increased self-determination [13]. Research studies conducted previously demonstrated how vagus nerve stimulation affects psychological health and social performance [14]. The success of our study faces several constraints despite the existing promising data. The 12-week study duration potentially fails to demonstrate the long-term maintenance of nVNS seizures reduction since the data gathers limited data points. Longitudinal studies need to be conducted because they will demonstrate how well therapeutic results last across multiple time periods [15]. Since the sham stimulation appeared and functioned equally to active nVNS use there is a chance that placebo effects were involved. Future research needs to incorporate additional objectiveo-metry tests like electroencephalography (EEG) recordings to substantiate observed improvements. The research showed similar mild adverse effect rates including headache and temporary neck pain when compared to studies about non-invasive vagus nerve stimulation [16]. All procedures demonstrated good safety outcomes which demonstrate the safe nature of this technique. The assessment of device usability together with patient tolerance requires expansion across different patient groups for researchers to establish broader generalization of their findings [17]. The therapeutic benefit of non-invasive vagus nerve stimulation can be improved by finding optimized settings for frequency along with intensity and duration parameters [18].

## Conclusion:

External application of vagus nerve stimulation (nVNS) presents itself as an innovative therapy which lowers seizure episodes and enhances life quality among epilepsy patients who do not respond to medications. The safe nature of nVNS along with its user-friendly application establishes it as a strong alternative to present-day epilepsy treatments. Additional extensive research including longer follow-ups must be conducted to verify long-term therapeutic outcomes while optimizing stimulation parameters for the purposes of clinical implementation.
